# The bornavirus-derived human protein EBLN1 promotes efficient cell cycle transit, microtubule organisation and genome stability

**DOI:** 10.1038/srep35548

**Published:** 2016-10-14

**Authors:** Katie N. Myers, Giancarlo Barone, Anil Ganesh, Christopher J. Staples, Anna E. Howard, Ryan D. Beveridge, Sarah Maslen, J. Mark Skehel, Spencer J. Collis

**Affiliations:** 1 Genome Stability Group, Sheffield Institute for Nucleic Acids (SInFoNiA), Academic Unit of Molecular Oncology, Department of Oncology & Metabolism, University of Sheffield Medical School, Beech Hill Road, Sheffield S10 2RX, UK; 2 Mass Spectrometry Group, The MRC Laboratory of Molecular Biology, Division of Cell Biology, Hills Road, Cambridge, CB2 0QH, UK

## Abstract

It was recently discovered that vertebrate genomes contain multiple endogenised nucleotide sequences derived from the non-retroviral RNA bornavirus. Strikingly, some of these elements have been evolutionary maintained as open reading frames in host genomes for over 40 million years, suggesting that some endogenised bornavirus-derived elements (EBL) might encode functional proteins. EBLN1 is one such element established through endogenisation of the bornavirus N gene (BDV N). Here, we functionally characterise human EBLN1 as a novel regulator of genome stability. Cells depleted of human EBLN1 accumulate DNA damage both under non-stressed conditions and following exogenously induced DNA damage. EBLN1-depleted cells also exhibit cell cycle abnormalities and defects in microtubule organisation as well as premature centrosome splitting, which we attribute in part, to improper localisation of the nuclear envelope protein TPR. Our data therefore reveal that human EBLN1 possesses important cellular functions within human cells, and suggest that other EBLs present within vertebrate genomes may also possess important cellular functions.

All viruses interact with cellular components of the host’s cells to facilitate their replication and infection cycles, and it has long been known that in addition to acquiring genes from their hosts, many retroviruses are capable of depositing endogenous viral elements into host genomes^1^,^2^,^3^. Over millions of years of viral infection, such integration events can accumulate, accounting for a significant proportion of the host organism’s genome^1^,^2^. Furthermore, during host evolution, endogenised retroviral elements can develop new functions within host cells. Although this is often associated with a role in inhibiting future viral infection^4^,^5^, some endogenised viral-derived elements can develop fundamental biological functions e.g. the essential role in mammalian placenta formation for the retroviral envelope gene-derived syncytin protein^6^,^7^.

Endogenisation of non-retroviral derived elements had not been observed in mammals until the recent discovery of endogenous bornavirus elements (EBLs) in multiple vertebrate species, including humans^8^,^9^,^10^,^11^,^12^. These studies also revealed that endogenous bornavirus integration events have occurred independently in many different species on multiple occasions, and have been maintained within the host’s genome for over 40 million years^8^,^10^,^12^,^13^. Intriguingly, some human EBLs contain potential protein encoding open reading frames and are detected in expressed sequence tag databases^8^,^10^. This discovery therefore raised the possibility that some EBLs may encode proteins that have developed fundamental biological roles within the host cell.

We recently carried out a human genome-wide siRNA-based screen to identify novel regulators of genome stability^14^,^15^,^16^,^17^. As part of our on-going analyses of candidates identified in this screen, the uncharacterised putative human gene LOC340900 was identified as a positive hit and therefore a potential novel genome stability factor. Following the discovery of EBLs in the human genome^8^,^10^, LOC340900 was subsequently renamed human EBLN1 as an EBL derived from the bornavirus N element. As no known biological function for human EBLN1, or indeed any endogenous bornavirus derived element had been determined, we carried out functional characterisation of human EBLN1.

Here we show that human EBLN1 facilitates a cell cycle transit and prevents the accumulation of both endogenous DNA damage and exogenously induced DNA damage. We also show that human EBLN1 is required for microtubule organisation and for preventing premature centrosome splitting, which we attribute, in part, to improper localisation of the nuclear envelope protein TPR. Our findings therefore demonstrate that akin to some retroviral-derived integrated elements, the evolutionary conserved endogenised bornavirus element EBLN1 has developed a functional role within human cells, and raises the possibility that other EBLs may have acquired biological functions within the host cell.

## Results

### Human EBLN1-depleted cells accumulate DNA damage

**Figure 1:**
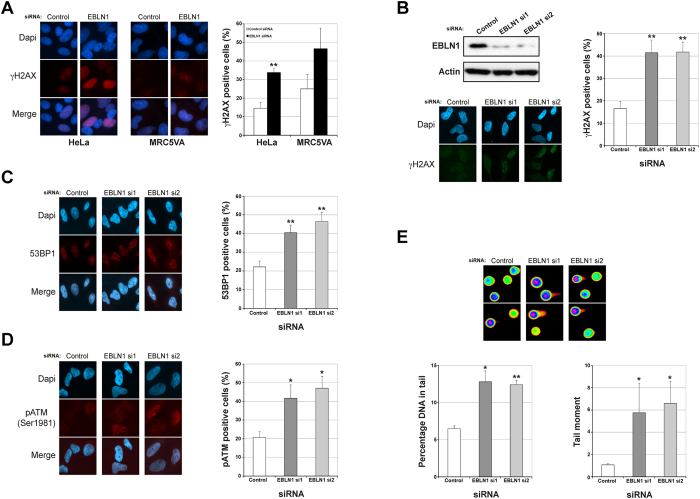
EBLN1-depleted cells accumulate DNA damage. (**A**) Left and middle panels show representative immunofluorescence images of γH2AX foci in HeLa and MRC5A cells respectively, with the right panel showing quantification of γH2AX foci. Data shown represents the mean from at least three independent experiments with associated SEMs (*p ≤ 0.05 and **p ≤ 0.01 compared to control siRNA cells). (**B**) Upper panel shows a representative western blot from HeLa cells demonstrating siRNA-mediated knockdown of EBLN1 with two individual siRNA. Additional examples of efficient EBLN1 depletion using these two siRNA are shown in Supplementary Figure S1A. Bottom panel shows representative γH2AX foci in control and individual EBLN siRNA-transfected HeLa cells, with the graph on right showing quantification of γH2AX foci. Data shown represents the mean from at least three independent experiments with associated SEMs (**p ≤ 0.01 compared to control siRNA cells). (**C**) Left panel shows representative immunofluorescence staining of 53BP1 foci in control and EBLN1-transfecetd HeLa cells. Graph on right shows mean percentage of 53BP1 positive cells. Data shown represents the mean from at least three independent experiments with associated SEMs (**p ≤ 0.01 compared to control siRNA cells). (**D**) Same as (**C**), except for pATM (Ser1981) foci in siRNA-transfected cells. Data shown represents the mean from at least three independent experiments with associated SEMs (*p ≤ 0.05 compared to control siRNA cells). (**E**) Upper panel shows representative images of COMETs in control and EBLN1 siRNA-transfected HeLa cells. Lower panels show quantification of mean percentage DNA in COMET tails and mean COMET tail moment. Data shown represents the mean from at least three independent experiments with associated SEMs (*p ≤ 0.05 and **p ≤ 0.01 compared to control siRNA cells).

We recently carried out a human genome-wide siRNA screen in HCT116 colorectal cancer cells to identify novel regulators of genome stability, employing increased γH2AX immunofluorescent foci as an established cellular marker of DNA damage^14^,^15^,^16^. Using this approach, human EBLN1 (originally designated LOC340900) was identified as a strong positive candidate (z-score = 1.93), which we subsequently validated in both HeLa and MRC5A cells using the same siRNA pool used in the HCT116-based screen (Fig. 1A). These findings are consistent with the identification of LOC340900 as a positive hit in a similar γH2AX-based siRNA screen carried out in HeLa cells^18^, and are comparable with z-scores within our screen of well-established DNA damage response, cell cycle checkpoint and genome stability factors such as CDC25A (2.15), CLSPN (2.05), RPA2 (2.02), Cyclin E (1.99), RFC1 (1.98) and WRN (1.95) amongst others. We next deconvolved the siRNA pool into 4 individual siRNA targeting EBLN1. All four EBLN1 targeted siRNA gave increased DNA damage, from which, two individual EBLN1-directed siRNAs that gave the most robust depletion of EBLN1 and led to comparable increases in γH2AX foci were selected to carry out the further functional characterisation of EBLN1 (Fig. 1B and Supplementary Figure S1A). In addition to a marked increase in γH2AX foci, depletion of EBLN1 by either siRNA also resulted in increased 53BP1 foci (Fig. 1C); an established cellular marker of DNA double-strand breaks (DSBs). Consistent with these data, EBLN1-depleted cells showed a marked increased phosphorylation of ATM kinase on serine 1981 (Fig. 1D), an activating event in response to DNA damage. Finally, to confirm that these cellular markers are due to a physical increase in DNA breaks within EBLN1-depleted cells, we carried out COMET assays to directly visualise DNA breaks. Compared to control siRNA-treated cells, EBLN1-depleted cells exhibited an approximately 2-fold increase in the number of DNA breaks (Fig. 1E), which is consistent with the increased numbers of γH2AX, 53BP1 and pATM foci, and comparable with the z-score of 1.93 obtained from the original siRNA-based γH2AX screen.

**Figure 2:**
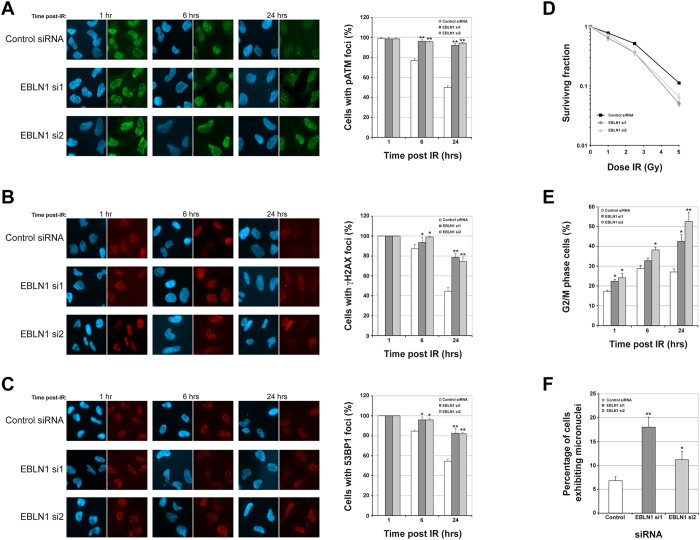
EBLN1-depleted cells fail to adequately repair exogenously induced DNA damage. (**A**) Left panel shows representative immunofluorescence images for phosphorylated ATM (pATM Ser1981) in HeLa cells transfected with either control of two individual EBLN1 siRNA at the indicated times post 3Gy IR. The graph on the right shows mean percentage of pATM foci positive cells in control and EBLN1 siRNA transfected cells. Data shown represents the mean from at least three independent experiments with associated SEMs (**p ≤ 0.01 compared to control siRNA cells). (**B**) Same as (**A**), but for γH2AX positive cells. Data shown represents the mean from at least three independent experiments with associated SEMs (*p ≤ 0.05 and **p ≤ 0.01 compared to control siRNA cells). (**C**) Same as (**B**), but for 53BP1 foci positive cells. Data shown represents the mean from at least three independent experiments with associated SEMs (*p ≤ 0.05 and **p ≤ 0.01 compared to control siRNA cells). (**D**) Clonogenic survival curves for control and EBLN1 siRNA-transfected HeLa cells treated with the indicated doses of IR. Data shown represents the mean surviving fractions with their respective SEMs calculated from at least three independent experiments. Comparable data was obtained in MRC5A cells (Supplementary Figure S1B). (**E**) FACS-based quantification (PI staining) of G2/M cells in control and EBLN1 siRNA-transfected HeLa cell populations at the indicated times post 3Gy IR. Data shown represents the mean from at least three independent experiments with associated SEMs (*p ≤ 0.05 and **p ≤ 0.01 compared to control siRNA cells). **(F**) Quantification of micronuclei in HeLa cells, as assessed by DAPI immunofluorescence staining, forty-eight hours post-transfection with either control or EBLN1 siRNA. Data shown represents the mean from at least three independent experiments with associated SEMs (*p ≤ 0.05 and **p ≤ 0.01 compared to control siRNA cells).

Given that these findings, we sought to determine if EBLN1-depleted cells responded efficiently to exogenously induced DNA damage. Ionising radiation (IR) treated EBLN1-depleted cells exhibited a comparable DNA damage response signalling compared with control siRNA-transfected cells, as evidenced by the initial phosphorylation induction of the DNA damage signalling kinase ATM (Fig. 2A). However, unlike control siRNA-transfected cells, a high level of activated ATM was still present in EBLN1-depleted cells 24 hours post-IR (Fig. 2A), indicative of unresolved DNA lesions and/or persistent checkpoint activation. Consistent with this notion, EBLN1-depleted cells also exhibited increased amounts of γH2AX and 53BP1 foci compared with control siRNA-transfected cells at late time points following IR (Fig. 2B,C respectively), exhibited increased IR sensitivity (Fig. 2D and Supplementary Figure S1B), post-IR induced G2/M delay, and a heightened amount of micronuclei compared with cells transfected with control non-targeting siRNA (Fig. 2E,F respectively). Collectively, these data suggested that DNA damage accumulates in EBLN1-depleted cells through an inability to appropriate resolve DNA lesions.

### EBLN1-depleted cells exhibit cell cycle abnormalities

**Figure 3:**
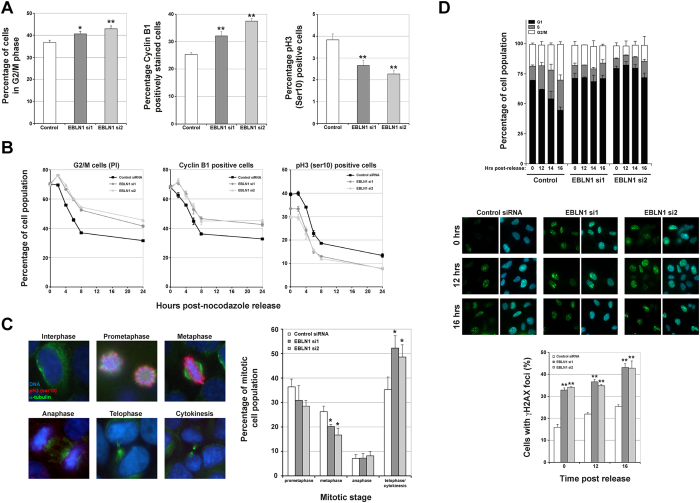
Cell cycle abnormalities in EBLN1-depleted cells. (**A**) Left, middle and right panels show respective FACS-based quantification of G2/M cells (propidium iodide staining), Cyclin B1 and phosphorylated Histone H3 (Ser10) positively stained HeLa cells in asynchronous cell populations transfected with the indicated siRNA. Data shown represents the mean from at least three independent experiments with associated SEMs (*p ≤ 0.05 and **p ≤ 0.01 compared to control siRNA cells). (**B**) Same as in (**A**), but for cell populations arrested with nocodazole and released back into the cell cycle and indicated markers quantified at the indicated time points post-nocodazole release. EBLN1 siRNA transfected cells exhibited a statistically significant difference compared with control siRNA-transfected cells (p < 0.05 for G2/M cells and p < 0.01 for cyclin B1 and pH3 positive cells). (**C**) Left panel shows representative immunofluorescence staining of DAPI, phosphorylated histone H3 (Ser10) and alpha tubulin to highlight specific mitotic phases that were scored in siRNA-transfected HeLa cells. Right panel shows quantification of these mitotic phases in control and EBLN1 siRNA-transfected cells 4 hours post-nocodazole release. The percentage of mitotic cells as assessed by DAPI-stained mitotic bodies in asynchronous and in populations following 4 hours post-nocodazole release are shown in Supplementary Figures S1E and S1F respectively. Data shown represents the mean from at least three independent experiments with associated SEMs (*p ≤ 0.05 and **p ≤ 0.01 compared to control siRNA cells). (**D**) Upper panel shows cell cycle distributions of RPE-1 cells transfected with either control or two independent EBLN1 siRNA at the indicated times following release from serum starvation. Middle panel shows representative immunofluorescence staining for γH2AX for the indicated cell populations with the graph below showing the average number of γH2AX positive cells within the siRNA-transfected populations at the indicated time points. Data shown is from two independent experiments with associated SEMs (**p ≤ 0.01 compared to control siRNA cells).

The heightened DNA damage in EBLN1-depleted cells, and the established links between DNA damage response and the cell cycle promoted us to determine if depletion of EBLN1 had any deleterious effects on cell cycle progression. Propidium iodide DNA staining in conjunction with FACS-based analyses was used to generate quantitative cell cycle profiles in control and EBLN1 siRNA-transfected cell populations. Depletion of EBLN1 gave a modest but consistent increase in the number of cells residing in the G2/M phase of the cell cycle (Fig. 3A and Supplementary Figure S1C), which is consistent with recent findings following knockdown of human EBLN1 in an oligodendroglia cell line^19^. To disseminate this population further, we assessed G2 and M phase populations in control and EBLN1 siRNA-treated cells using Cyclin B1 and phosphorylated histone H3 on Ser10 (pH3 Ser10) respectively. Compared to control siRNA-transfected cells, EBLN1-depleted cells exhibited increased populations of Cyclin B1 positive and decreased populations of pH3 Ser10 positive cells (Fig. 3A and Supplementary Figure S1D). In keeping with theses findings, we also observed an increased number of mitotic cells in EBLN1-depleted cell populations (Supplementary Figure S1E), suggesting that transit through mitosis might be perturbed in EBLN1-depleted cells.

We therefore enriched siRNA-transfected cells for early mitotic populations using nocodazole and analysed cell cycle transit using PI, Cyclin B1 and pH3 Ser10 levels following nocodazole removal. Compared with control siRNA treated cells, EBLN1-depleted cells exhibited an initial increase in both the G2/M population and Cyclin B1 levels (Fig. 3B), suggesting either an inefficient release from nocodazole block, or an accumulation of late G2/early mitotic cells from cell populations that were initially arrested during the nocodazole treatment. As cells progressed through mitosis and into the next cell cycle, EBLN1-depleted cells exhibited a comparable rate in the reduction of Cyclin B1 levels to that observed in control siRNA-transfected cells (Fig. 3B). However, they consistently consisted of more Cyclin B1 positive cells at each time point post-nocodazole release compared with control siRNA-treated cells (Fig. 3B). In contrast, pH3 ser10 levels were always lower in EBLN1-depleted cells in both nocodazole-blocked cells and following resumption of cell cycle transit (Fig. 3B). These data therefore prompted us to study the mitotic transit of EBLN1-depleted cells in more detail by direct visualisation of cells using immunofluorescence detection of DNA (DAPI), pH3 ser10 and α-tubulin to assess chromosome condensation and separation as cells progressed through mitosis. Compared with control siRNA transfected cells, EBLN1-depleted cells exhibited a greater proportion of cells in late stages of mitosis such as telophase and cytokinesis (Fig. 3C), which is consistent with an overall increase in mitotic bodies observed in nocodazole blocked/released EBLN1-depleted cells (Supplementary Figures S1F), and a reduced growth rate in cells depleted of EBLN1 (Supplementary Figure S1G). To determine if these cell cycle abnormalities contributed to the increased DNA damage in EBLN1-depleted cells, or vice versa, we carried cell cycle synchronisation/release time course experiments. As observed in asynchronous cell populations, G0/G1 enriched, as well as post-release EBLN1-depleted cells exhibited increased amounts of DNA damage compared with control siRNA treated cells (Fig. 3D). Interestingly, unlike control siRNA treated cells that quickly re-entered cell cycle following release from serum starvation, EBLN1-depleted cells exhibited a severe delay in re-entering the cell cycle, which may be due to the higher amounts of DNA damage prevalent within these cells (Fig. 3D). Collectively, these data reveal that EBLN1-deficient cells exhibit cell cycle transit defects under normal growth conditions and in response to exogenously-induced cell cycle pausing.

### EBLN1-depleted cells exhibit microtubule and centrosomal defects

**Figure 4:**
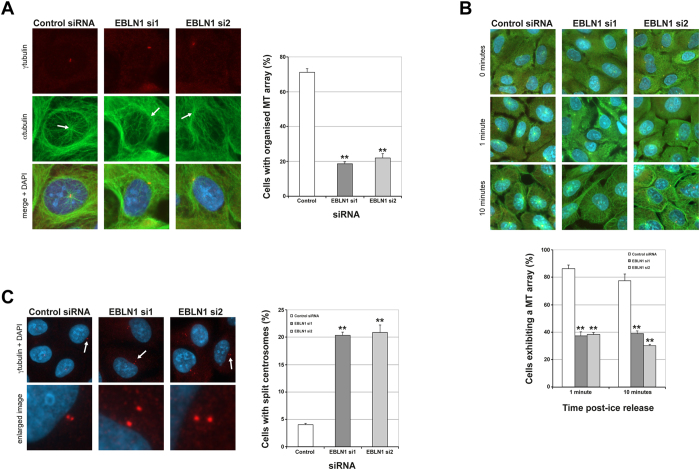
EBLN1 deficiency leads to microtubule and centrosomal defects. (**A**) Left panel; representative immunofluorescence images for γtubulin (centrosomes), αtubulin (microtubules) and merged images with DAPI (nucleus), in interphase U2OS cells transfected with either control or two individual EBLN1 siRNA. The white arrows on the αtubulin images highlight the position of the centrosome from which a microtubule array should emanate. Right panel; quantification of organised microtubule (MT) arrays in interphase U2OS cells treated with either control or EBLN1 siRNA as indicated. Data shown represents the mean from three independent experiments with associated SEMs (*p ≤ 0.05 and **p ≤ 0.01 compared to control siRNA cells). (**B**) Upper panel shows representative merged immunofluorescence images for γtubulin, αtubulin and DAPI at the indicated times points post release from ice treatment. The graph below shows mean percentage of control and EBLN1 siRNA transfected U2OS cells exhibiting an organised microtubule array. Data shown represents the mean from at least three independent experiments with associated SEMs (**p ≤ 0.01 compared to control siRNA cells). (**C**) Left panel shows representative merged immunofluorescence images for γtubulin and DAPI in U2OS cells transfected with the indicated siRNA. The white arrows highlight the centrosomes that are shown in the enlarged image below. Right panel shows the mean percentage of cells exhibiting split centrosomes in control and EBLN1 siRNA transfected (>2 microns apart). Data shown represents the mean from at least three independent experiments with associated SEMs (*p ≤ 0.05 and **p ≤ 0.01 compared to control siRNA cells).

Microtubules are critical for regulating cell growth as well as cell division processes through association with centrosomes, which are the main microtubule organising centres. As such, defects in microtubule organisation and centrosome number/duplication processes can lead to genome instability^20^. Therefore, given the increased genome instability and cell cycle defects observed in EBLN1-depleted cells, we sought to determine if EBLN1 is important for microtubule architecture. As previously described^14^,^21^, interphase U2OS cells transfected with control siRNA displayed an organised radial array of microtubules emanating from the centrosome (Fig. 4A). However, depletion of EBLN1 led to a marked loss of microtubule organisation (Fig. 4A), indicating that EBLN1 is important for maintaining a stable microtubule array. This is consistent with our findings that GFP-tagged EBLN1 resides mainly within the cytoplasm, likely due to the lack of the nuclear localisation sequences found in BDV N^10^,^22^, and is occasionally localised to microtubule structures (Supplementary Figure S1H), although this could be partly due to trafficking within the cell.

We next assessed if microtubule organisation during regrowth was affected in EBLN1-depleted cells following rapid depolymerisation by treatment with ice^14^,^21^. Unlike control siRNA-transfected cells that rapidly regrew an organised microtubule network following release from ice treatments (Fig. 4B), cells depleted of EBLN1 exhibited an approximate 50% reduction in the proportion of cells exhibiting an organised microtubule array at comparable time points (Fig. 4B). Given that both abnormal centrosome numbers as well as centrosomal separation defects can give rise to microtubule abnormalities and genome instability^20^,^23^,^24^, and the microtubule defects and heightened genome instability present associated with EBLN1 deficiency, we next assessed if centrosomal defects were evident in EBLN1-depleted cells. The most striking feature of EBLN1-depleted cells was a marked increase in centrosome splitting (Fig. 4C). This phenotype is unlikely to be a consequence of large changes to cell cycle positioning, as this is not evident in EBLN1-depleted cells (Fig. 3A and Supplementary Figure S1C), and is a much smaller distance to that associated with cells in G2 phases of the cell cycle^25^, suggesting that reduced levels of EBLN1 impact on microtubule organisation and regrowth.

### EBLN1 interacts with TPR, but not with the Cyclin B1-CDK1 complex

**Figure 5:**
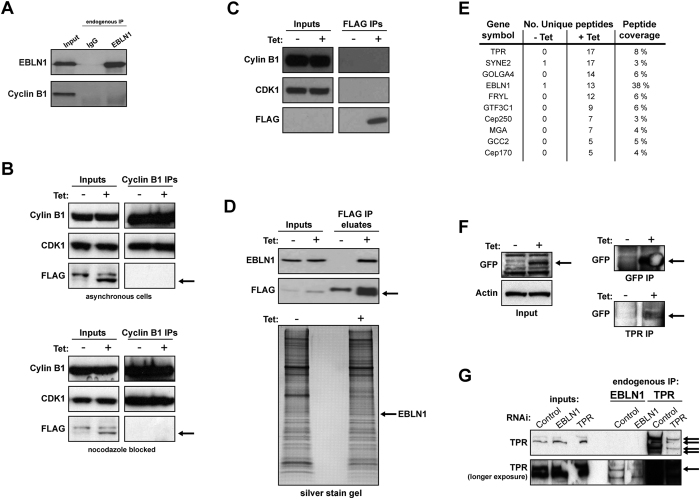
EBLN1 does not interact with the Cyclin B1-CDK1 complex, but does interact with TPR. (**A**) Immunoprecipitation of endogenous EBLN1 from HeLa cells probed with either EBLN1 or Cyclin B1 antibodies. Agarose beads incubated with cell extracts and IgG was used as a negative control for non-specific protein binding. Black arrow indicates FLAG-tagged EBLN1 band. (**B**) Immunoprecipitation (IP) of endogenous Cyclin B1 from asynchronous (upper panel) or mitotic (lower panel) FLAG-EBLN1 expressing HeLa cells probed with the indicated antibodies. The minus tetracycline (−Tet) samples (uninduced expression of FLAG-EBLN1) serve as negative controls for non-specific protein binding. (**C**) Immunoprecipitation of FLAG-EBLN1 from tetracycline-inducible HeLa cells probed with the indicated antibodies. Minus tetracycline samples serve as negative controls for non-specific protein binding. (**D**) Upper panel shows EBLN1 and FLAG western blots of eluates from FLAG-EBLN1 expressing tet-inducible HeLa cells. Lower panel shows a SYPRO Ruby stained polyacrylamide gel of FLAG eluates shown in the upper panel. Black arrow indicates FLAG-tagged EBLN1 band. (**E**) Table showing some of the most prevalent proteins co-immunoprecipitating with FLAG-EBLN1 as determined by proteomic analyses of the eluates shown in (**E**). The number of unique peptides for each protein is shown for both uninduced and induced (−Tet and + Tet) samples to highlight enrichment in FLAG-EBLN1 eluates (+Tet samples), along with the respective peptide coverage for each protein identified. (**F**) Indicated western blots on inputs (left panel), GFP immunoprecipitations and TPR immunoprecipitations (right panels) in stable tetracycline-inducible GFP-EBLN1 expressing HeLa cell lines. Arrows highlight GFP-EBLN1 band in each IP. Note that a longer exposure is shown for the TPR IPs compared with the GFP IPs. (**G**) TPR western blots of immunoprecipitated endogenous TPR from HeLa cells transfected with the indicated siRNA. Arrows highlight TPR isoforms (upper panel) and TPR-specific band in EBLN1 IPs (lower panel), which are reduced in cells transfected with TPR siRNA. Note that the TPR antibody is not capable of recognising endogenous TPR in the input lanes, only purified TPR in the IP lanes.

Previous work has suggested that the bornavirus N gene (BDV N), from which human EBLN1 is derived, interacts with Cyclin B1-CDK1 complex within mammalian cells, which may be important for viral infection and propagation^26^. Given the role of Cyclin B1-CDK1 in controlling mitotic transit and the cell cycle abnormalities observed in EBLN1-depleted cells, we hypothesised that EBLN1 may also be binding to the Cyclin B1-CDK1 complex to facilitate normal mitotic transit. However, immunoprecipitation of endogenous EBLN1 failed to detect any interaction with endogenous Cyclin B1 (Fig. 5A). Likewise, Cyclin B1 or CDK1 did not co-purify with FLAG-tagged EBLN1 following induced expression in stable cell lines (Fig. 5B,C respectively).

To identify potential interactors of human EBLN1 as a means to help elucidate a possible mechanistic role in cell division and microtubule organisation/re-growth processes, we carried out proteomic analyses of purified FLAG-EBLN1 complexes using a tetracycline inducible cell line system (Fig. 5D). Consistent with the cell division and microtubule organisation/re-growth phenotypes observed in EBLN1-depleted cells, several potential EBLN1 integrators we identified have known biological roles in the organisation and regulation of microtubules e.g. TPR, FRYL, SYNE2, CEP250 and CEP170 (Fig. 5E). Of particular interest were TPR (Translocated Promoter Region) and FRYL (Furry Homolog-Like). TPR is a nuclear basket protein that regulates mRNA transport through nuclear pore complexes^27^, and has also been shown to promote efficient mitotic progression through the regulation of mitotic spindles^28^. FRYL is the human homologue of the drosophila Furry protein that regulates the actin cytoskeleton^29^. Through a number of experimental approaches, we were unable to verify any interactions between ELBN1 and FRYL (data not shown). However, we were able to detect an interaction between GFP-tagged EBLN1 and endogenous TPR (Fig. 5F), as well as a weak but reproducible interaction between endogenous EBLN1 and at least one isoform of endogenous TPR (Fig. 5G), suggesting that these two proteins may functionally interact within human cells.

### Functional interplay between EBLN1 and TPRN

**Figure 6:**
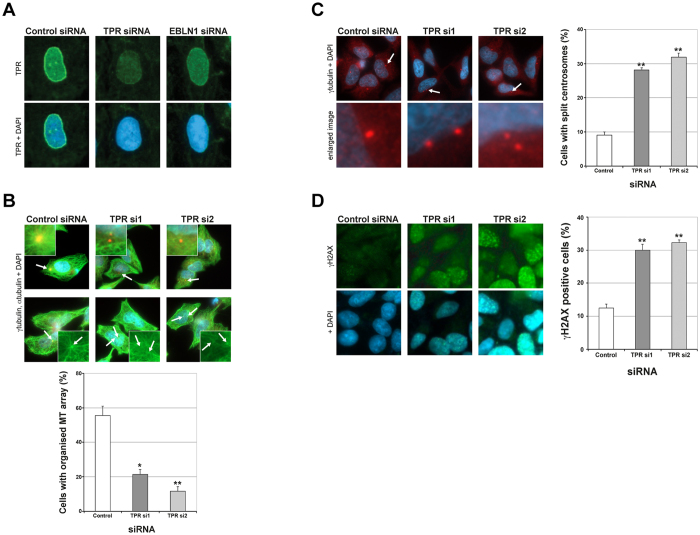
Functional interplay between EBLN1 and TPR. (**A**) Representative immunofluorescence images showing endogenous TPR staining in interphase U2OS cells transfected with the indicated siRNA. Additional examples are given in Supplementary Figure S1I. (**B**) Upper panel; representative immunofluorescence images of microtubule organisation (αtubulin staining; green, γtubulin; red and DAPI; blue) in interphase U2OS cells treated with either control or TPR siRNA as indicated. White arrows indicate the centrosomes (microtubule organising centres) that are shown enlarged in the inset images. Lower panel; quantification of organised microtubule (MT) arrays in interphase U2OS cells treated with either control or TPR siRNA. Data shown represents the mean from three independent experiments with associated SEMs (*p ≤ 0.05 and **p ≤ 0.01 compared to control siRNA cells). (**C**) Left panel; representative immunofluorescence images for centrosomes (γtubulin; red) in control and TPR siRNA treated U2OS cells. White arrows indicate the centrosomes shown in the enlarged images below. Right panel; quantification of centrosome splitting defects observed in cells treated with either non-targeting control or TPR siRNA. Data shown represents the mean from at least three independent experiments with associated SEMs (**p ≤ 0.01 compared to control siRNA cells). (**D**) Left panel; representative immunofluorescent images of γH2AX in control non-targeting and TPR siRNA treated U2OS cells, with graph on right showing average number of γH2AX positive cells in each siRNA transfected population. Data shown represents the mean from at least three independent experiments with associated SEMs (*p ≤ 0.05 and **p ≤ 0.01 compared to control siRNA cells).

Given our interaction data, we next assessed if depletion of EBLN1 could impact TPR function. Depletion of EBLN1 caused disruption to the nuclear envelope localisation of TPR (Fig. 6A and Supplementary Figure S1J), which was not a consequence of reduced TPR expression levels (Supplementary Figure S1I). Given this finding, and that TPR has been previously shown to regulate microtubules^28^, we reasoned that the microtubule defects observed in EBLN1-depleted cells could be a consequence of disrupted TPR function due to its mis-localisation. We therefore assessed microtubule organisation and centriolar distance in TPR-depleted cells. Similar to our findings for EBLN1-depleted cells, knockdown of TPR by two independent siRNA led to disrupted microtubule organisation in both interphase cells and during regrowth following acute disruption (Fig. 6B). Furthermore, depletion of TPR led to premature centrosome splitting phenotype, which was comparable to that observed in EBLN1-depleted cells (Fig. 6C). Finally, we determined that cells depleted of TPR accumulate DNA damage in a similar manner to that observed in EBLN1-depleted cells (Fig. 6D). This is in keeping with the fact that TPR was identified as a positive hit in both our γH2AX screen (z-score of 1.8), and in a comparable screen carried out by the Cimprich laboratory^18^. Collectively these data provide compelling evidence that human EBLN1 has a biological function within human cells to facilitate TPR localisation at the nuclear envelope, promote a stable microtubule array and suppression of DNA damage.

## Discussion

Endogenisation of viral genes into the genome of the host organism can give rise to retained viral-derived protein-encoding elements that possess advantageous biological functions^1^,^2^,^3^. Such biologically active roles of endogenised viral elements are normally associated with reducing subsequent viral infection^4^,^5^. However, through evolutionary processes, endogenised viral elements can develop new and important biological functions within their original host’s genomes^6^,^7^. The recent discovery of 40 million year old endogenised bornavirus elements (EBLs) that comprised an ORF structure, and EST detection within the genomes of many vertebrates raised the possibility that some EBLs may have an active biological function^8^,^10^,^11^,^12^,^13^,^30^. Indeed, it has recently been demonstrated that a ground squirrel EBLN is capable of inhibiting bornavirus replication and infection^9^, although a molecular understanding of how it may promote such biological functions remains unknown.

We show here that human EBLN1 is important for preventing the accumulation of endogenous DNA damage, as well as for the efficient repair of exogenously induced DNA damage. Furthermore, we reveal that EBLN1-depleted cells exhibit abnormal cell cycle progression (specifically in resolving the final stages of mitosis), which are likely a consequence of increased amounts of DNA damage along with microtubule and centrosomal splitting defects. This is particularly intriguing given the established links between centrosome dynamics and genome instability^20^,^23^,^24^, and that disruption to microtubules and/or premature centrosome splitting can affect chromosomal segregation dynamics^31^,^32^. In keeping with a model based on our data that these phenotypes are in part due to improper localisation and function of TPR in EBLN1-depleted cells; we show that TPR-depleted cells exhibit comparable microtubule and centrosomal defects to that observed in EBLN1-depleted cells, as well as increased amounts of DNA damage. Furthermore, it has previously been shown that disruption to TPR function through siRNA-mediated depletion causes chromosomal segregation defects^28^, and we show here that EBLN1-depleted cells exhibit increased numbers of micronuclei, a common consequence of mitotic division defects^33^, and a phenotype previously associated with deficiency in TPR^28^.

Intriguingly, TPR is one of only a few nuclear pore complex proteins that have been implicated in cancer development^27^,^34^. Given that genome instability is a defined hallmark of cancer cells^19^, our findings raise the possibility that disruption to EBLN1 function could play a potential role in the development and/or progression of human disease such as cancer. Analysis of the current Cancer Genome Atlas reveals that although no EBLN1 mutations have so far been identified in human tumours, a small percentage of tumour types exhibit EBLN1 amplification or deletion. This is interesting given our depletion data presented here, and more so by our attempts to rescue EBLN1-associated phenotypes using a combination of UTR-directed siRNA and ectopic expression of EBLN1, which in itself led to increased amounts of DNA damage (Supplementary Figure S2A). Interestingly, a similar phenotype was not observed in cells ectopically expressing BDV N, from which EBLN1 is derived (Supplementary Figure S2B), which also failed to rescue cellular phenotypes associated with reduced human EBLN1 levels, thus suggesting that human EBLN1 has gained additional and important biological functions within human cells during the many millions of years that it has resided within our genomes. In conclusion, our data represents the first detailed molecular and functional characterisation of a human endogenised bornavirus element, and raises the possibility that other EBLs within vertebrate genomes may also possess important biological functions.

## Materials and Methods

### Cell culture

HeLa, MRC5VA, U2OS, RPE-1 and HEK293 and cells were maintained as adherent monolayer cultures in DMEM media containing 10% FBS and 1% penicillin/streptomycin at 37 °C in a humidified atmosphere of 5% CO_2_. HeLa Flp-in T-REx and HEK293 Flp-In T-REx cells (Invitrogen) were maintained in DMEM media containing 10% FBS and 1% penicillin/streptomycin, supplemented with 4 μg/ml Blasticidin S (Melford) and 100 μg/ml Zeocin (Invitrogen).

### FACS analyses & cell cycle profiling

Cell populations were washed in PBS and fixed overnight in 70% ethanol at −20 °C. Fixed samples were washed 3 times in PBS before treatment with 5 μg of RNAse1 followed by the addition of 300 μl of Propidium Iodide (50 μg/ml) to each sample. FACS acquisition was carried out using a FACS-Calibur (Becton-Dickinson) and analysed by FlowJo (Tree Star, Inc). On average, 20,000 live cells were gated and quantified for each sample, with each condition replicated 2–3 times for each individual experiment. Statistically significant differences between cell populations were confirmed from at least 3 independent experiments using a 2-tailed t-test, assuming equal variances. For nocodazole block/release experiments, cells were treated with 100 ng/ml for 16 hours and synchronisation confirmed by FACS analyses. For release experiments, nocodazole-containing media was removed, cells washed in PBS and fresh pre-warmed media added. Circa 60,000 RPE-1 cells were seeded into one well of a 6 well plate per sample (on cover slips for immunofluorescence) and supplemented with growth media. To arrest the cells in G1/G0, the normal growth media was replaced with serum-free media and transfected with 0.5 μM siRNA per sample, which then remained serum starved for ~60 hrs. Synchronised serum-starved cell populations were then released into to growth media with the first sample was harvested 10 hours post release and then every 2 hours thereafter.

### EBLN1 siRNA depletion

Briefly, 4 μl Dharmafect 1 was mixed with 50 μM siRNA and 1.2 × 10^4^ cells were seeded into one well of a 6-well plate and transfected. This ratio was scaled appropriately depending on cell number and the surface area utilised. Cells were harvested at either 48 or 72 hours post treatment. siRNA sequences (Dharmacon On Target Plus) were as follows (all 5′ to 3′): Control siRNA: UAAUGUAUUGGAACGCAUA, EBLN1 siRNA 1: CCGCUGUUGUGUUGGAGAU, EBLN1 siRNA 2: UAGCUAUAAUGCAGGGCCA, EBLN1 UTR: ACGUGAACUAAUCCUUAUAUU, TPR siRNA 1: GAAGAAGUGCGUAAGAAUA and TPR siRNA 2: UCAGUUGACUCCAGGAAUA.

### Stable cell line production

Full-length EBLN1 was cloned from genomic DNA from HeLa cells and BDV N was cloned from a pCDFDuet-1 plasmid containing full-length BDV N. Each gene was cloned into the Gateway entry vector pDONR221 (Life Technologies) following a PCR reaction with the following primer sets; EBLN1_F: GGGGACAAGTTTGTACAAAAAAGCAGGCTTGTCCCGCCCAAGAAACAAC and EBLN1_R:GGGGACCACTTTGTACAAGAAAGCTGGGTTTATTCAAATCCCGAAATCCC or BDV N_F: CTTTATGCGACTCCTGATTAGG and BDV N_R: CGGCCGATATCCAATTGAGAT. Purified pDONR221-EBLN1 and pDONR221-BDV N were independently mixed with LR clonase and either FLAG-pDEST/FRT/TO or GFP-pDEST/FRT/TO to generate FLAG- or GFP-tagged tetracycline inducible stable HeLa and HEK293 cell lines as previously described^35^. All plasmids were fully sequence verified using a range of external and internal sequencing primers.

### Cell lysis and western blotting

Cell lysates were prepared by solubilising cell populations on ice for 20 minutes in lysis buffer; 50 mM Tris-HCl pH 7.5, 150 mM NaCl, 1% Triton X-100, 1 mM DTT and 1 mM EDTA supplemented with 50 U/μl benzonase (Novagen), protease and phosphatase inhibitors (Sigma). Cleared lysates were produced by centrifugation of the resulting samples at 16,000 × g for 15 min at 4 °C. Gel electrophoresis was performed using the NuPAGE system (Invitrogen). Briefly, samples were resolved on 4–12% Bis-Tris gels in MOPS buffer, transferred to a PVDF membrane which was then probed for the protein of interest using antibodies diluted in TBS containing 5% Marvel and 0.1% Tween-20 (Sigma). Antibodies used were: alpha-Tubulin (Abcam ab7792, 1:1000), pATM (ab36810), β-actin (Abcam; ab8226), Cyclin B1 (Cell Signaling; mAb4135), EBLN1 (in-house synthesis and purification of a rabbit polyclonal antibody through Sheaf Innovations/BioServUK Ltd. using the peptide MSRPRNNPQTSSPQD), FLAG (Sigma; F3165), FLAG-HRP (Sigma; A8592), GFP (Abcam; ab290), anti-Histone H3 pSer10 (Cell Signaling; 9701), γH2AX (Cell Signalling; 25775), securin (Abcam; ab79546), TPR (ab58344, ab170940 and ab70610), HRP-secondary antibodies (DAKO, FITC) and Alexa-Fluor antibodies (Invitrogen).

### Immunoprecipitation and proteomic analyses

For purification of FLAG-tagged proteins, 1 mg of a whole-cell extract was incubated with 20 μl of M2-anti FLAG beads (Sigma) for 16 hours at 4 °C. For immunoprecipitation using endogenous antibodies, 1–2 μg of antibody was incubated with the sample for 1–2 hours before addition to 20 μl of washed Protein A/G beads (Santa Cruz) and incubation for 16 hours at 4 °C. Beads were then pelleted and washed three times in 20x dry bead volumes of lysis buffer. The bound protein was eluted either by heating the beads at 95 °C for 5 min with 2x LDS buffer (Invitrogen) or by incubation with FLAG peptide (Sigma) according to manufacturer’s instructions. Inputs represent ~1/20th of the extract used for the immunoprecipitation. For proteomic analyses, protein samples were reduced, alkylated and digested with trypsin, using the Janus liquid handling system (PerkinElmer, UK). The digests were subsequently analysed by LC-MS/MS on an LTQ Orbitrap XL mass spectrometer, (ThermoScientific, San Jose, USA). LC-MS/MS data were searched against a protein database (UniProt KB) using the Mascot search engine programme and all subsequent data were interrogated manually.

### Immunofluorescence

1.2 × 10^3^ cells were seeded onto 200 mm glass cover slips in 6 well plates and incubated for 24 hrs for the cells to adhere. Following appropriate siRNA treatments and/or drug treatments, cells were fixed in either ice-cold 100% methanol or 4% PFA for 10 minutes (depending on the antibody used). Coverslips were washed briefly in PBS and extracted with 3% BSA, 0.2% Triton-X100 dissolved in PBS for 30 minutes, followed by a further brief wash in PBS. Antibodies were diluted in PBS containing 1% BSA at optimised concentrations and a 100 μl aliquot added to each cover slip for 1 hour. This was followed by 3 × 5 minutes washes in PBS. Secondary Alexa-Fluor antibodies (1:500) and DAPI (1 μg/ml) were diluted in PBS containing 1% BSA and a 100 μl aliquot added to each cover slip for 1 hour. This was followed by 3 × 5 minutes washes. Processed coverslips were mounted onto glass slides with 10 μl Shandon immuno-mount (Thermo). Microtubule array assays were carried out as previously described^14^. All immunofluorescence images were captured on a Nikon Eclipse T200 inverted microscope (Melville), equipped with a Hamamatsu Orca ER camera, a 200 W metal arc lamp (Prior Scientific,UK) and an 60x objective lens. Images were captured and analysed using Volocity 3.6.1 software (Improvision) and exported as tiff files. Scoring for each individual condition (siRNA, cell line, drug treatment etc.) within an experiment was carried out on at least 10 separate fields of view containing between 200–300 cells in total. Cells were scored positive for DDR foci if they contained >5 discrete foci per nucleus and the mean percentage of positive cells from at least 3 independent experiments were then calculated and plotted along with their respective standard errors of the means (SEM). Statistically significant differences between cell populations was confirmed using a 2-tailed t-test, assuming equal variances and are presented on figures as *p ≤ 0.05, **p ≤ 0.01.

### Quantitative PCR

RNA was extracted from cells using a Qiagen RNEasy Plus Kit and quantified on a Nanodrop spectrophotometer. Equal quantities of RNA (2 μg) were then reverse transcribed using an Applied Biosystems High Capacity RNA- cDNA kit as per the manufacturer’s instructions. Resulting cDNAs were diluted as needed to carry out 10 μl Taqman reactions in triplicate using an Applied Biosystems Taqman Gene Expression Master Mix and Applied Biosystems 7900HT Thermal cycler using the manufacturer’s recommended conditions and analysed, which were subsequently analysed using Applied Biosystems SDS 2.4 software to calculate respective Δct values and relative gene expression levels. Taqman probes used were GAPDH; Hs02758991_g1, EBLN1; Hs00908304_s1 and TPR; Hs00162918_m1.

### COMET assays

Alkaline COMET assays were performed using the Trevigen kit system according to the manufacturers’ protocol. Briefly, cells were re-suspended at 1 × 10^5^ per ml in low melting point agarose and transferred to the COMET slide. After setting, slides were incubated for 30 min in lysis solution, and then incubated in unwinding solution for 20 min. Slides were then electrophoresed at 21 volts for 30 min before rinsing twice in water and once in 70% ethanol. After drying, cells were stained with SYBR Green and visualised by fluorescence microscopy. COMET tail moments were analysed from at least 100 cells per condition using COMETScore software and from at least 3 independent experiments. Statistically significant differences between cell populations were confirmed from at least 3 independent experiments using a 2-tailed t-test, assuming equal variances.

### Cytotoxicity and clonogenic survival assays

For MTT cytotoxicity assays, cells were plated at a density of between 1000–2000 cells/well in 96-well plates (dependant on cell line used), and the following day transfected with appropriate siRNA, and drug added 2 days later at various concentrations. After 5 days of growth, MTT reagent was added to the cells at a final concentration of 3 mg/ml, and incubated at 37 °C for 3 hrs. The media was removed and replaced with 200 μl DMSO to solubilise the formazan product, which was quantified by determining optical density at 540 nm using a spectrophotometric microtitre plate reader. Cytotoxicity was calculated for each treatment by normalisation to appropriate vehicle only controls for each set of transfectants. For clonogenic survival assays, 500–5000 cells were plated onto 100 mm dishes in triplicate 4 hour prior to treatment with ionising radiation (IR) to allow cells to attach. When colonies could be observed in the control plates (10–14 days post-IR), cells were fixed and stained with methylene blue in methanol (4 g/L), and colonies consisting of more than 50 cells were subsequently scored. Surviving fractions (SF) were calculated based on the plating efficiency (fraction of colonies formed in untreated plates) for each cell population/treatment as; SF = (No. cells counted)/(No. cells plated x plating efficiency).

## Additional Information

**How to cite this article**: Myers, K. N. *et al*. The bornavirus-derived human protein EBLN1 promotes efficient cell cycle transit, microtubule organisation and genome stability. *Sci. Rep.* **6**, 35548; doi: 10.1038/srep35548 (2016).

## Supplementary Material

Supplementary Information
