# Supplementary material for: The bornavirus-derived human protein EBLN1 promotes efficient cell cycle transit, microtubule organisation and genome stability
Source: Sci Rep. 2016 Oct 14;6:35548. doi: 10.1038/srep35548 (PMC5064366; doi:10.1038/srep35548)
Supplement: Supplementary Information [file srep35548-s1.pdf]

Supplementary Figure legends and Figures for:

The bornavirus-derived human protein EBLN1 promotes efficient cell cycle transit, microtubule organisation and genome stability.

Katie N. Myers[1*], Giancarlo Barone[1*], Anil Ganesh[1], Christopher J. Staples[1#], Anna E. Howard[1], Ryan D. Beveridge[1], Sarah Maslen[2], J. Mark Skehel[2], & Spencer J. Collis[1*].

Supplementary Figure S1.

A: Representative western blots in U2OS (upper panel) and RPE-1 cells (lower panel) transfected with either control or two individual EBLN1-targetting siRNA. Actin blots serve as a loading control. **B**: Clonogenic survival curves for control and EBLN1 siRNA-transfected MRC5A cells treated with the indicated doses of IR. Data shown represents the mean surviving fractions with their respective SEMs calculated from at least three independent experiments. **C**: Left and right panels show FACS-based quantification (PI) of cell cycle phases respectively in HeLa or MRC5A cells transfected with the indicated siRNA. Data shown represents the mean from at least three independent experiments with associated SEMs (*$p \leq 0.05$ compared to control siRNA cells). **D**: Western blot showing reduced phosphorylated histone H3 (Ser10) in asynchronous EBLN1 siRNA-transfected cells, which is complementary to the data shown in Figure 3B. **E**: Quantification of mitotic cells (DAPI stained bodies assess by immunofluorescence) in asynchronous cell populations. Data shown represents the mean from at least three independent experiments with associated SEMs (*$p \leq 0.05$ compared to control siRNA cells). **F**: Same as in (D), but in cell populations following 4 hours following release from an overnight nocodazole treatment. Note that data shown in Figures S1E and S1F are complementary to that shown in Figure 3C. Data shown represents the mean from at least three independent experiments with associated SEMs (*$p \leq 0.05$ compared to control siRNA cells). **G**: Growth curves (MTT) for cells treated with either control of EBLN1 siRNA assessed over the indicated time period. Data shown represents the mean surviving fractions with their respective SEMs calculated from at least three independent experiments. These data are consistent with recent findings of reduced growth rates following knockdown of human EBLN1 in an oligodendroglia cell line[1]. **H**: Representative immunofluorescence detection of GFP-tagged EBLN1 in tetracycline inducible expressing stable cell lines. The observed cyctoplasmic localisation of human EBLN1 is consistent with that observed in human oligodenroglioma cells transiently transfected with a plasmid expressing HA-tagged human EBLN1[2]. **I**: Additional immunofluorescence images showing endogenous TPR staining in U2OS cells transfected with the indicated siRNA as shown in Figure 6A. **J**: Quantification of EBLN1 and TPR mRNA levels by RT-PCR 48hrs post-transfection with the indicated siRNA. Percentage knockdown of

target mRNA (normalised to GAPDH levels) were calculated compared to non-targeting control siRNA transfected cells, and data shown is the average from two independent experiments with their respective SEMs.

Supplementary Figure S2.

A: Upper panel; representative immunofluorescence images of γH2AX staining in tetracycline-inducible GFP-EBLN1 expressing cells as indicated. Lower panel; quantification of the percentage of cells exhibiting >10 γH2AX foci per cell in the indicated cell populations. Data shown represents the mean from at least three independent experiments with associated SEMs (**$p \leq 0.01$ compared to control siRNA cells). **B**: Left panel; representative western blot showing tetracycline-inducible expression of GFP-tagged BDV N. Cells were treated with either control non-targeting siRNA or siRNA targeting human EBLN1 as indicated to show specificity towards human EBLN1. Right panel; quantification of the percentage of cells exhibiting >10 γH2AX foci per cell in the indicated cell populations. . Data shown represents the mean from at least three independent experiments with associated SEMs (**$p \leq 0.01$ compared to control siRNA cells). Note that the high background observed in the -Tet control siRNA treated cells can occasionally occur in these cells and has been previously reported[3].

Myers et al - Supplementary Figure S1

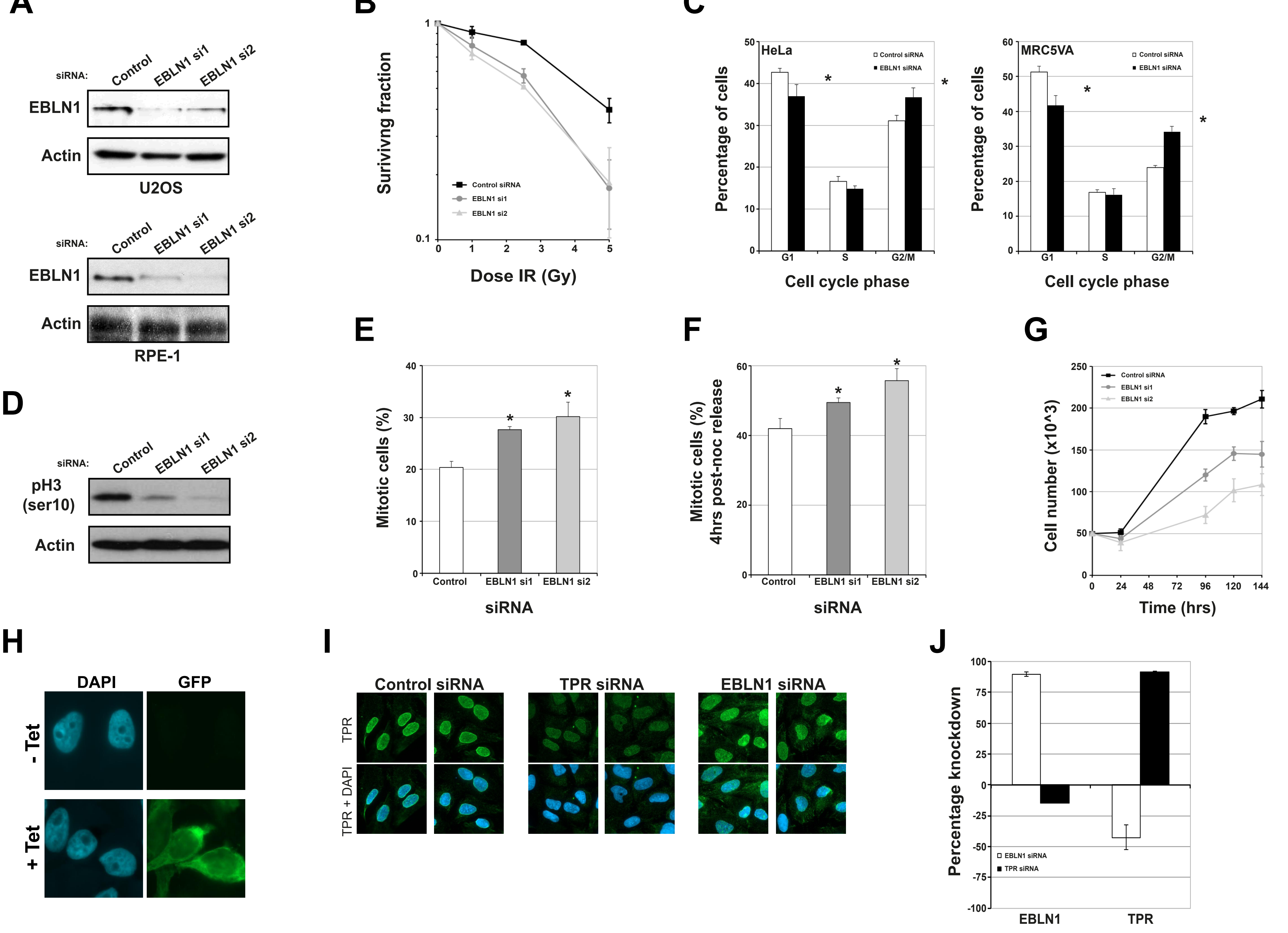

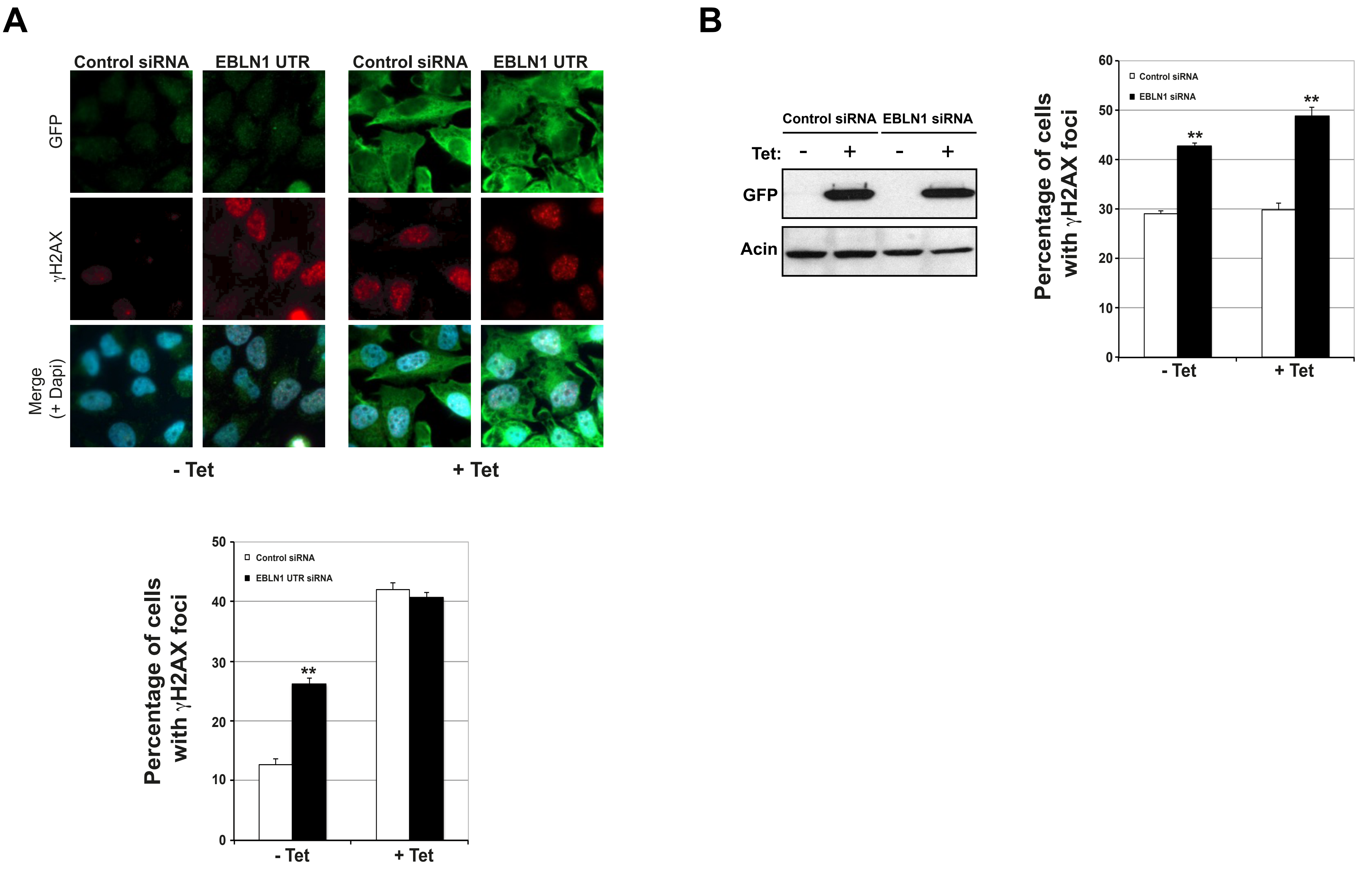

Myers et al - Supplementary Figure S2
